# Enhanced Effective Connectivity From Ipsilesional to Contralesional M1 in Well-Recovered Subcortical Stroke Patients

**DOI:** 10.3389/fneur.2019.00909

**Published:** 2019-08-21

**Authors:** Yanmin Peng, Jingchun Liu, Minghui Hua, Meng Liang, Chunshui Yu

**Affiliations:** ^1^Department of Radiology and Tianjin Key Laboratory of Functional Imaging, Tianjin Medical University General Hospital, Tianjin, China; ^2^School of Medical Imaging, Tianjin Medical University, Tianjin, China

**Keywords:** brain infarction, motor cortex, motor recovery, magnetic resonance imaging, diffusion tensor imaging

## Abstract

**Background and Purpose:** Interhemispheric imbalance may provide a framework for developing new strategies to facilitate post-stroke motor recovery especially for patients in chronic stage. Using effective connectivity analysis, we aimed to investigate interactions between the bilateral primary motor cortices (M1) and their correlations with motor function and M1-related structural and functional changes in well-recovered patients with chronic subcortical ischemic stroke.

**Methods:** Twenty subcortical stroke patients and 20 normal controls underwent multimodal magnetic resonance imaging (MRI) examinations. During the movement of the affected hand, functional MRI was used to calculate the M1 activation and M1-M1 effective connectivity. Diffusion tensor imaging was used to compute the fractional anisotropy (FA) of the affected corticospinal tract (CST) and M1-M1 anatomical connection. After intergroup comparisons, we tested whether the altered M1-M1 effective connectivity was correlated with the motor function, M1 activation and FA of the affected CST and M1-M1 anatomical connection in patients.

**Results:** Compared to normal controls, stroke patients exhibited increased excitatory effective connectivity from ipsilesional to contralesional M1 and increased ipsilesional M1 activation; however, they showed reduced FA values in the affected CST and M1-M1 anatomical connection. The increased effective connectivity was positively correlated with motor score and the FA of the M1-M1 anatomical connection, but not with the M1 activation or the FA of the affected CST in these patients.

**Conclusions:** These findings suggest that the enhancement of M1-M1 effective connectivity from ipsilesional to contralesional hemisphere depends on the integrity of the underlying M1-M1 anatomical connection (i.e., less deficits of the M1-M1 anatomical connection, greater enhancement of the corresponding effective connectivity), and such M1-M1 effective connectivity enhancement plays a supportive role in motor function in chronic subcortical stroke.

## Introduction

Motor disability is one of the most common deficits after stroke. Post-stroke motor recovery depends mainly on structural damage and functional reorganization of the motor network. The aim of motor rehabilitation is to enhance the beneficial functional reorganization, which could be identified by observing structural and functional changes during spontaneous recovery. In the past decades, neuroimaging studies, especially those with magnetic resonance imaging (MRI), have revealed a variety of structural, functional, connectivity and network changes in the brain after stroke (1–3). However, the clinical importance of these changes depends on their relations to motor recovery and possibilities to be integrated into therapeutic strategies.

In patients with subcortical stroke, structural, and functional changes of the primary motor cortex (M1) have been related to motor recovery. Although more extensive activation in both hemispheres are commonly observed in stroke patients, only normalized ipsilesional M1 activation has been consistently related to motor recovery (4). As the main descending fibers of the M1, the corticospinal tract (CST) damage has been identified as the main cause for motor deficit and the leading barrier for motor recovery (5, 6). Besides the lesion-induced direct damage in the CST, the integrity of transcallosal fibers between the bilateral M1 is also reduced in subcortical stroke (7, 8), which has been correlated with the bilateral recruitment of motor areas (8) and the increase in M1-M1 functional connectivity (9). As a simple method to assess interhemispheric functional interactions, the resting-state functional connectivity between the bilateral M1 experiences a reduction and recovery process, and the normalized or enhanced connectivity has been related to motor recovery (10, 11). In contrast to the lack of directionality of the resting-state functional connectivity, the effective connectivity measures the influence of one brain area exerts over another, can better characterize interhemispheric functional interactions, providing useful information for planning rehabilitation strategies (2).

The model of interhemispheric imbalance is the basis for developing non-invasive brain stimulation (NIBS) strategies to facilitate post-stroke motor recovery (12). On the basis of inhibitory influence from contralesional to ipsilesional M1 (2, 12), these strategies mainly aim to increase ipsilesional M1 excitability and/or reduce contralesional M1 excitability to recover the balance (13, 14). However, inconsistent therapeutic effects on stroke patients (15, 16) indicate the diversity of interhemispheric interactions and the existence of unknown modulators. The diversity of interhemispheric interactions has also been revealed by effective connectivity studies. For example, in patients with subcortical stroke, one study shows additional inhibitory influences from contralesional to ipsilesional M1 (17); however, another study demonstrates a positive influence (18). In addition, M1-M1 effective connectivity changes in subcortical stroke are not isolated from other structural damages and functional reorganization; instead, they are possibly related to structural and functional changes of the M1 (19)—this information is useful in the stratification of patients for suitable interventions.

Although early interventions may be most beneficial by preventing the development of maladaptive reorganization and resulting in greater motor improvement, interventions for chronic stroke patients are also important because a huge number of chronic stroke patients with persistent motor disabilities are waiting for new strategies to improve their impaired motor functions. Examining the relationship between functional reorganization and structural impairment in chronic stroke patients with well recovered motor function may help clarify the beneficial role of the functional reorganization in motor recovery in chronic stage (9, 20). For example, it has been reported that chronic stroke patients with well recovered motor function show reduced cortical thickness but increased task-evoked activation and resting-state neural activity (i.e., regional homogeneity and amplitude of low-frequency fluctuation) and functional connectivity in the ipsilesional M1 (20). Furthermore, in a similar group of patients, we observed that the FA values of the anatomical connections between bilateral M1 and of the CST were reduced but the M1-M1 resting-state functional connectivity was increased, and interestingly, the resting-state functional connectivity was positively correlated with the FA values of these anatomical connections (9). However, it is unclear whether effective connectivity between bilateral M1 plays a similar beneficial role in motor recovery in these patients. Here, we aimed to identify stroke-induced M1-M1 effective connectivity changes in relatively well recovered subcortical stroke patients in chronic stage, and to investigate their functional roles in motor recovery and associations with the ipsilesional M1 activation and the white matter integrity of the affected CST and M1-M1 anatomical connections. Importantly, we directly evaluated the relationship between the M1-M1 effective connectivity and the motor function of these patients.

## Materials and Methods

### Subjects

Twenty well-recovered chronic stroke patients with subcortical infarcts participated in this study. All patients satisfied the inclusion criteria of first-ever ischemic stroke, clear motor deficits at the time of stroke onset, single subcortical lesion involving the motor pathway, right-handed before the stroke [determined using the Chinese edition of the Edinburgh Handedness Inventory (21)], an interval of more than 6 months from stroke onset, well-recovered in motor function with Fugl-Meyer Assessment (FMA) score more than 60 for the affected upper extremity and more than 90 for the whole extremities, and capable of completing neurological and MRI examinations. The exclusion criteria included (1) recurrent stroke, (2) any other brain abnormalities, (3) lacunes and microbleeds based on T1-, T2-, and diffusion-weighted images (DWI), (4) severe white matter hyperintensity manifested as a Fazekas scale (22) score > 1, (5) serious cerebral atrophy and (6) a history of drug dependency or psychiatric disorders. Twenty age-, sex-, and handedness-matched normal volunteers were recruited as controls. This study was approved by the Ethics Committee of Tianjin Medical University General Hospital and informed consent was obtained from each participant before the study. Parts of these participants have been used in our previous work (9, 20).

### Task Design

All subjects performed a block-design motor task (a unilateral voluntary hand-grasping task) with a frequency of 2.4 Hz. The frequency of the task was controlled by a computer. An experimenter inside the scanner room could see the signal on the computer monitor and would lightly touch the foot of the participant. Each participant was instructed to close his fist once perceiving a light touch on the foot. All participants were trained to perform this task until they could perform well the task prior to the formal experiment. Patients with stroke performed the task using the affected hand, but healthy controls used their left hand. Each task block (20 s) was followed by a resting block (20 s), and the cycle was repeated four times. Instructions for the start and the end of each block were given by a visual cue on a screen. The detailed procedures for the task were described previously (20).

### Image Acquisition

MRI data were acquired using a Signa HDx 3.0-Tesla scanner (General Electric, Milwaukee, WI). The functional MRI (fMRI) data were acquired by a gradient-echo single-shot echo-planar imaging (SS-EPI) sequence: repetition time/echo time (TR/TE) = 2,000/30 ms; field of view (FOV) = 240 mm × 240 mm; matrix = 64 × 64; slice thickness = 3 mm; 1 mm gap; 38 interleaved transversal slices. The diffusion tensor imaging (DTI) data were obtained using a spin-echo SS-EPI sequence with 30 non-collinear diffusion-sensitized directions and a *b*-value of 1,000 s/mm^2^, and with 3 sets of *b*=0 images. The parameters were TR/TE = 11,000/77.6 ms; FOV = 256 mm × 256 mm; matrix = 128 × 128; slice thickness = 3 mm, no gap; and 50 transversal slices. DWI and conventional MR images (T1- and T2 -weighted images) were acquired for brain abnormality assessment. DWI was obtained using the following imaging parameters: TR/TE = 3,000/61 ms; FOV = 240 mm × 240 mm; matrix = 160 × 160; slice thickness = 6 mm; gap = 1.5 mm; and b = 1,000 s/mm^2^. Sagittal 3D T1-weighted images were acquired by a brain volume sequence with the following imaging parameters: TR/TE = 7.8/3.0 ms; FOV = 256 × 256 mm; matrix = 256 × 256; inversion time=450 ms; flip angle= 13°; slice thickness=1 mm, no gap; and 176 slices.

### Image Preprocessing

To facilitate the analysis across patients with lesions on different sides, the imaging data were flipped from left to right along the midline for patients with left-sided lesions. Thus, the right side of the image corresponded to the ipsilesional hemisphere and the left side to the contralesional hemisphere for all patients.

The preprocessing of fMRI data was performed using the Statistical Parametric Mapping (SPM8, https://www.fil.ion.ucl.ac.uk/spm). The volumes were corrected for the acquisition time delay between slices and were then realigned across volumes to correct for inter-scan movements. We controlled for head motion with thresholds of 2.5 mm translation in each cardinal direction and 2.5° rotation around each orthogonal axis. The realigned fMRI images were spatially normalized to Montreal Neurological Institute (MNI) space using the EPI template and were then re-sampled to 3 × 3 × 3 mm^3^ voxels. The resulting images were smoothed with a Gaussian kernel of 8 mm full-width at half-maximum. A high-pass filter with 128-s cut-off was applied to eliminate signal drifts of each voxel. Head motion effects on fMRI signals were further reduced by regressing out the six head motion parameters from the fMRI time series of each voxel.

The FSL software (https://fsl.fmrib.ox.ac.uk/fsl/fslwiki/) was used for DTI data preprocessing, including Eddy-current distortion correction, head motion correction, skull removal, and FA calculation for each voxel in the whole brain. Then, Diffusion Toolkit (http://www.trackvis.org/dtk/) was used to track fiber tract of the CST and the M1-M1 fiber tract. Because the white matter integrity can be accurately assessed by FA only in fiber tracts with highly coherent arrangement (6), we only extracted the FA value of the cerebral peduncle for the affected CST and that of the midsagittal slice for the M1-M1 anatomical connection for subsequent analyses. More details about the processing of DTI data were described in our previous study (9).

### Task fMRI Analysis

We first created an ipsilesional M1 mask and a contralesional M1 mask as the Brodmann Area 4 in the corresponding hemisphere defined by the Broadmann area atlas available in the software package MRIcron (https://people.cas.sc.edu/rorden/mricron/) for the following brain activation analysis and Granger causality analyses. For the brain activation analysis, general linear model (GLM) was used to identify hand motion-induced activation map of each subject, and the contrast maps of all individuals were entered into a two-sample *t*-test to identify voxels within the ipsilesional and contralesional M1 masks that were activated differently between the two groups (*P* < 0.001, uncorrected). The identified voxels with significant intergroup activation difference located within the ipsilesional M1 mask were defined as the seed region for the subsequent effective connectivity analysis. The average activation (i.e., the beta values) of these identified voxels were also extracted from each participant for the subsequent correlation analyses.

### Granger Causality Analysis

Granger causality is a widely-used effective connectivity approach to explore the causal relationships between two time series based on their temporal precedence of each other (23). Here, the activity in brain area X can be considered to cause the activity in brain area Y if the blood-oxygen-level dependent (BOLD) signal of the current time point in brain area Y can be predicted using those of the past time points in brain area X. In this study, Granger causality was performed using the bivariate linear autoregressive model implemented in the software package REST (http://www.restfmri.net/forum/REST-GCA). In brief, Granger causality from brain area X to brain area Y can be estimated using the following equation:

(1) $$Y_{t}=\sum_{i=1}^{P}A_{i}X_{\left(t-i\right)}+\sum_{i=1}^{P}B_{i}Y_{\left(t-i\right)}+\;CZ_{t}+\varepsilon_{t}$$

where, *X*_*t*_ and *Y*_*t*_ represent the fMRI signals at the time point *t* in brain areas *X* and *Y*, respectively; *Z* represents covariates (e.g., head motion and global trend); *A*_*i*_ and *B*_*i*_ represent path coefficients and auto regression coefficients at the time lag *i*. The time series *X* significantly Granger causes the time series *Y* if the path coefficient *A*_*i*_ is significantly larger or smaller than zero for at least one time lag. The maximal time lag *p* represents the model order. As most previous studies (24), we set lag *p* as 1 in the present study.

*A*_*i*_ can be standardized to Z scores according to the following formula:

(2) $$\begin{array}{r} Z_{i}=\frac{A_{i}-m}{s} \end{array}$$

where *m* is the global mean (i.e., the mean of all voxels within the whole brain) of *A*_*i*_, and *s* is the corresponding standard deviation of *A*_*i*_. Negative path coefficients *A*_*i*_ mean inhibitory effects and positive path coefficients *A*_*i*_ mean excitatory effects (25).

To investigate Granger causality from the ipsilesional M1 seed region to contralesional M1 voxels, in formula (1), let *X* be the average time series of the ipsilesional M1 seed region and *Y* be the time series of a given voxel of the contralesional M1 mask (i.e., Brodmann Area 4), and repeat this estimation for all voxels within this contralesional M1 mask. Therefore, we obtained a map in which the value of each voxel in the contralesional M1 represents the Granger causality value from the seed to this particular voxel. Similarly, Granger causality from every contralesional M1 voxel to the seed can also be obtained by letting *X* be the time series of a given voxel and *Y* be the time series of the seed.

The voxel-wise group differences in Granger causality of each direction within the contralesional M1 mask were identified using a two-sample *t*-test under the threshold of *P* < 0.05 (corrected by voxel-level family wise error, FWE) in SPM8. Finally, the M1-M1 effective connectivity of each direction was represented by the average Granger causality values across all voxels showing significant intergroup differences within the contralesional M1 mask and used in the subsequent correlation analyses.

### Statistical Analysis

The average effective connectivity, the average activation, and the FA values of the affect CST and the M1-M1 anatomical connection were compared between patients and controls using a two-sample *t*-test (*P* < 0.05). Pearson's correlation analyses, after controlling for age, sex, lesion volumes and post-stroke interval, were performed to examine the relationships of the altered M1-M1 effective connectivity with the upper-limb FMA score, the activation of the ipsilesional M1, and the FA of the affected CST and M1-M1 anatomical connection in stroke patients (*P* < 0.05, uncorrected). The normality of these data was checked by one-sample Kolmogorov-Smirnov test. These analyses were performed using an in-house script in MATLAB (R 2015b).

## Results

### Demographic and Clinical Information

**Figure 1:**
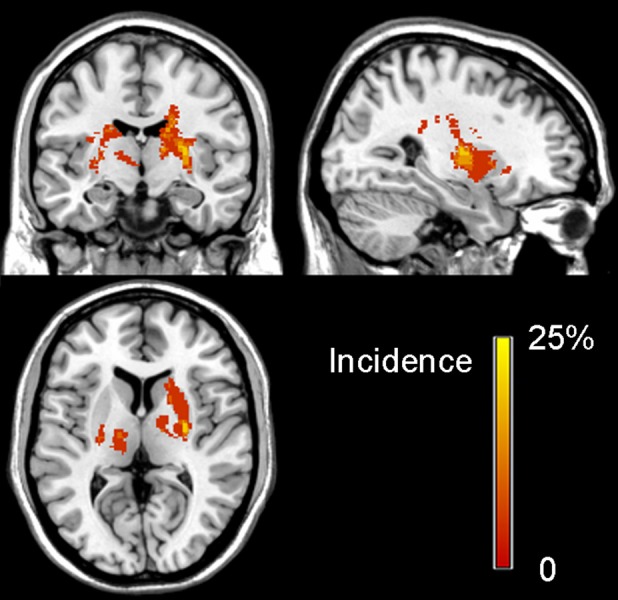
Lesion incidence map of patients with stroke in the chronic stage. The color bar denotes the probability of lesion distribution.

The clinical and demographic data of stroke patients and normal controls are listed in Table 1. Compared with normal controls, patients with stroke did not show any significant differences in age (*P* = 0.907) and sex (*P* = 0.900). The duration from stroke onset to the MRI scan ranged 11–64 months (mean value: 31.6 ± 16.54 months). The stroke lesions involved the internal capsule and the surrounding structures such as the thalamus, basal ganglia, and corona radiata; 9 out of 20 patients (i.e., 45%) had infarct lesions in the left hemisphere and 11 (i.e., 55%) in the right hemisphere. The lesion volume (mean ± standard deviation) is 1077.75 ± 1255.48 mm^3^ (range: 250–5694 mm^3^) and the lesion location is shown in Figure 1. The motor function of the patients was significantly recovered with an FMA > 93/100 for the whole extremities.

**Table 1:** Demographic and clinical information of patients with stroke and controls.

**Variables**	**Stroke** **patients (*n* = 20)**	**Normal controls** **(n = 20)**	***P*-value**
Age (year)	57.6 ± 8.5 (42–72)	57.3 ±7.5 (47–74)	0.907
Men, *n* (%)	12 (60%)	11 (55%)	0.900
Duration (months)	31.6 ± 16.5 (11–64)		
Lesion volume (mm^3^)	1077.75 ± 1255.48 (250–5694)		
Lesion location, *n* (%)			
Left hemisphere	9 (45%)		
Right hemisphere	11 (55%)		
Fugl-Meyer assessment			
Upper extremity	65.4 ± 1.0 (62–66)		
Whole extremity	98.9 ± 2.0 (93–100)		

*Data are presented as mean ± SD (range) for continuous data and n (%) for categorical data*.

### Intergroup Comparisons of Imaging Measures of Interest

**Figure 2:**
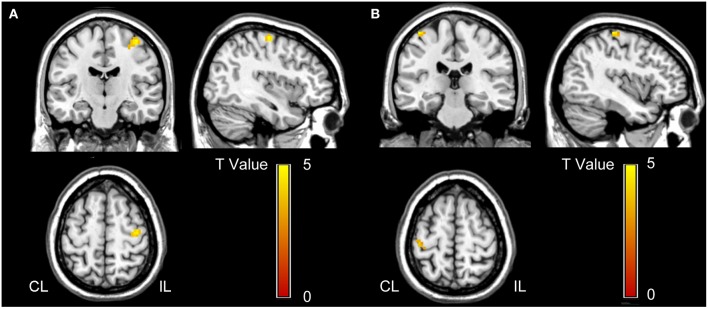
Voxel-wise comparisons in task-evoked activation **(A)** and effective connectivity **(B)** between stroke patients and normal controls. **(A)** Stroke patients show increased activation in the ipsilesional M1 than normal controls (*P* < 0.001, uncorrected). This cluster is used as the seed for the effective connectivity analysis. **(B)** Stroke patients demonstrate increased effective connectivity from ipsilesional to contralesional M1 than normal controls (*P* < 0.05, FWE corrected). CL, contralesional; FWE, family wise error; IL, ipsilesional; and M1, primary motor cortex.

The voxel-wise comparisons of the hand motion-induced activation within the bilateral M1 masks revealed a cluster in the ipsilesional M1 with significant activation difference between the two groups (peak coordinates = [39, −15, 63]; peak z score = 4.20; cluster size=28 voxels; *P* < 0.001, uncorrected; Figure 2A).

With this cluster as the seed, the voxel-wise Granger causality analysis showed that stroke patients exhibited significantly increased effective connectivity from the seed (the ipsilesional M1) to a cluster of voxels in the contralesional M1 (peak coordinates = [−39, −30, 66]; peak z score = 4.61; cluster size = 12 voxels; *P* < 0.05, FWE corrected; Figure 2B) compared to normal controls. However, we did not find any significant intergroup differences in the effective connectivity from contralesional to ipsilesional M1.

**Figure 3:**
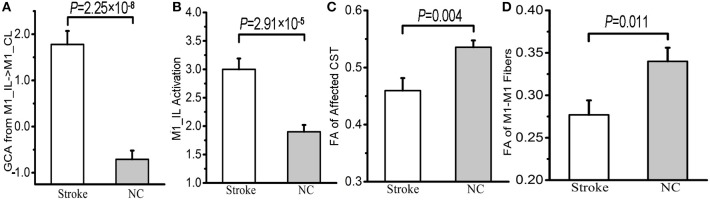
Differences in the effective connectivity from ipsilesional to contralesional M1 **(A)**, the activation of ipsilesional M1 **(B)**, the FA values of the affected CST **(C)** and M1-M1 anatomical connection **(D)** between stroke patients and normal controls. BOLD, blood oxygen-level dependent; CL, contralesional; CST, corticospinal tract; FA, fractional anisotropy; GCA, Granger causality analysis; IL, ipsilesional; M1, primary motor cortex; and NC, normal control.

The average effective connectivity extracted from the voxels within the identified contralesional M1 cluster was significantly increased in patients (shifted from inhibitory in controls to excitatory in patients) (*P* = 2.25 × 10^−8^; Figure 3A). The average activation (i.e., the beta values) of the ipsilesional M1 cluster (i.e., the seed region) were also significantly increased in patients compared with controls (*P* = 2.91 × 10^−5^; Figure 3B). In contrast, both the FA values in the affected CST (*P* = 0.004; Figure 3C) and the FA values of the M1-M1 anatomical connection were significantly decreased in patients (*P* = 0.011; Figure 3D).

### Correlations of M1-M1 Effective Connectivity With Motor Function and Other M1-Related Imaging Measures

**Figure 4:**
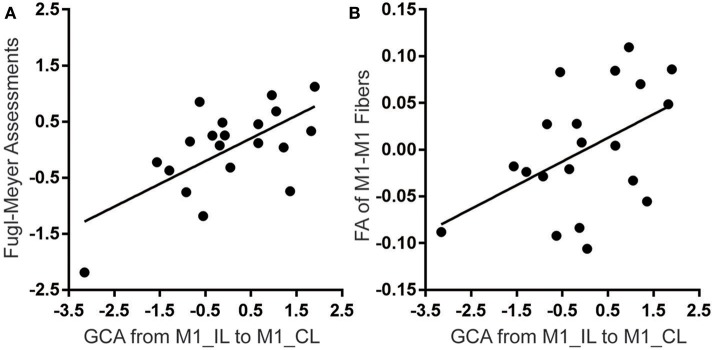
Significant correlations of the effective connectivity from ipsilesional to contralesional M1 with FMA score **(A)** and FA value of M1-M1 anatomical connection **(B)** in stroke patients. The y-axes represent residues of upper-limb FMA score or FA values after controlling for the effects of age, sex, lesion volumes and post-stroke interval. CL, contralesional; FA, fractional anisotropy; FMA, Fugl-Meyer assessment; GCA, Granger causality analysis; IL, ipsilesional; and M1, primary motor cortex.

When controlling for the effects of sex, age, lesion volume and post-stroke interval, we found that the strengths of the excitatory effective connectivity from ipsilesional to contralesional M1 were positively correlated with the upper limb FMA scores (correlation coefficient = 0.645; *P* = 0.002; Figure 4A) and with the FA values of the M1-M1 anatomical connection (correlation coefficient = 0.478; *P* = 0.033; Figure 4B) in patients with stroke. However, the strengths of this effective connectivity were not correlated with the FA values of the affected CST (correlation coefficient = 0.200; *P* = 0.399) or the activation of the ipsilesional M1 (correlation coefficient = −0.136; *P* = 0.569). After controlling for age, sex, lesion volumes, and post-stroke interval, these data are normally distributed, confirmed by Kolmogorov-Smirnov tests: *Z* = 0.452 (*P* = 0.987) for the M1-M1 effective connectivity, *Z* = 0.622 (*P* = 0.834) for the brain activation, Z = 0.451 (*P* = 0.987) for the FA values of the affected CST, and *Z* = 0.476 (*P* = 0.977) for FA values of the M1-M1 anatomical connection, *Z* = 0.759 (*P* = 0.613) for the upper limb FMA scores.

## Discussion

In this study, we investigated M1-M1 effective connectivity alterations and their associations with motor function and M1-related activation and connection changes in well-recovered subcortical stroke patients in the chronic stage. Note that, although all patients used in this study were in their chronic stage and well-recovered in motor function, they had clear and definite motor deficits at the time of stroke onset. Therefore, these patients represent a stroke population with an effective recovery in motor function and thus provide an essential basis for studying neural mechanisms underlying effective motor recovery in stroke patients. We found that the effective connectivity from ipsilesional to contralesional M1 was completely inverted from inhibitory in normal controls to excitatory in stroke patients, and the excitatory connectivity was positively correlated with motor function in these patients, indicating a supportive role in motor recovery. The positive correlation between the effective and anatomical connectivity of the bilateral M1 suggests that the enhancement of the M1-M1 effective connectivity depends on the integrity of the underlying anatomical connection.

In contrast to previous studies showing normalized M1-M1 effective connectivity (18), reduced inhibitory effective connectivity from ipsilesional to contralesional M1 (19), or increased inhibitory effective connectivity from contralesional to ipsilesional M1 (17) in the chronic stage of subcortical stroke, we found increased excitatory effective connectivity from ipsilesional to contralesional M1 in well-recovered patients with subcortical stroke in the chronic stage. It is noticeable that the patients examined in the previous studies and our present study were at different stages of motor recovery after stroke—Rehme et al. (18), Grefkes et al. (17) and our present study examined, respectively, patients at 2 weeks after stroke, patients with partial motor recovery in an early chronic stage after stroke, and patients with well-recovered motor function in a late chronic stage after stroke. Evidence has suggested that stroke patients at different stages may exhibit different functional changes in the brain. For example, using a rat model of stroke, van Meer (26) found that interhemispheric functional connectivity was reduced and associated with impaired motor performance in the first days after experimental stroke, and then was gradually increased and concomitant to sensorimotor improvements. All these findings suggest the complexity and diversity of interhemispheric functional interactions even in chronic stroke patients at different stages. Both well-recovered characteristic of patients and positive correlation between excitatory connectivity and motor performance support a beneficial role of the increased excitatory connectivity in motor recovery at least in chronic subcortical stroke patients with a good motor function. Our findings might also imply the importance of incorporating connectivity-based information into the design of more effective NIBS protocols which would be beneficial to the existing great number of stroke patients who have developed into the chronic stage (27, 28). Future studies are needed to identify the suitable NIBS protocols that could enhance the beneficial excitatory effective connectivity from ipsilesional to contralesional M1 to facilitate motor recovery.

The M1-M1 effective connectivity is calculated based on activities of the bilateral M1 during the movement of the affected hand. It is plausible to speculate a correlation between M1-M1 effective connectivity and M1 activation (19). However, we failed to find such a correlation, suggesting different functional meanings of the two measures in terms of motor function. The normalized activation in the ipsilesional M1 has been reported to be an indicator for better motor recovery (4, 17); however, the enhanced excitatory effective connectivity from ipsilesional to contralesional M1 was found to be an indicator for better motor function in the present study. Thus, further clarification of effects of modulating the M1 excitability with NIBS techniques on M1 activation and M1-M1 effective connectivity may help design an appropriate protocol for facilitating motor recovery.

It has been observed a reduced FA of M1-M1 transcallosal fibers in patients with subcortical stroke which has been considered to reflect the pathological process of Wallerian degeneration that is secondary to the direct damage of the CST by stroke lesions (7–9). Furthermore, the FA values of the compromised M1-M1 anatomical connection have found to be negatively correlated with the resting-state M1-M1 functional connectivity in stroke patients, indicating that the increased M1-M1 functional connectivity could compensate for the impaired anatomical connection to some extent (9). In this study, however, we found a positive correlation between the excitatory effective connectivity from ipsilesional to contralesional M1 and the FA of the M1-M1 anatomical connection, suggesting that the severe impairment of the M1-M1 anatomical connection may reduce the potential of the effective connectivity to be reorganized to compensate for motor deficit. Although both enhanced functional (9) and effective connectivity (this study) between the bilateral M1 are associated with motor recovery, they may have different functional meanings in terms of interhemispheric anatomical connection impairment. Our finding also indicates that the assessment of the white matter integrity of M1-M1 transcallosal fibers may be useful in screening patients with less impairment that are more likely to enhance the excitatory effective connectivity from ipsilesional to contralesional M1.

Several limitations in the present study should be noted. Considering differential effects of degree of recovery, post-stroke interval and lesion location on effective connectivity changes of the motor system (17–19), the inclusion of a group of homogeneous well-recovered subcortical stroke patients in the chronic stage in this study may help to identify reliable effective connectivity changes in these specific population of patients. However, our findings cannot be generalized to all stroke patients. Future studies should investigate the longitudinal effective connectivity changes in a large group of stroke patients with different lesion locations and motor deficits to discern the diversities of the connectivity changes. Although we investigated the influences of M1 activation and M1-related anatomical connection integrity on the increased excitatory effective connectivity from ipsilesional to contralesional M1, we did not explore the relationship between the excitability of the bilateral M1 and the effective connectivity, which may provide information on how to increase the effective connectivity by modulating the M1 excitability via NIBS strategies. Moreover, we included patients with lesions in either the right or the left hemisphere and images of the patients with left-side lesions were flipped in the analyses to increase the statistical power and to facilitate the comparison between patients and controls. However, this procedure might introduce bias to our results due to unmatched hand in the movement task between groups. Finally, this study only focused on the M1-M1 effective connectivity changes to answer the questions of our interest. However, stroke patients may also have effective connectivity changes within the motor system in the same hemisphere or between motor and non-motor systems, and thus systematic investigation of all possible effective connectivity changes after stroke may provide complete understanding of functional reorganization of the brain in stroke patients.

## Conclusion

In this study, we found a complete inversion of the effective connectivity from ipsilesional to contralesional M1 from inhibitory influence in normal controls to excitatory in stroke patients. The positive correlation between excitatory connectivity and motor function suggests that this excitatory connectivity may facilitate motor recovery. The dependency of the enhancement in this effective connectivity on the integrity of M1-M1 anatomical connection indicates that integrity assessment of M1-M1 connection may help to screen patients with greater potential to motor recovery through enhancing this excitatory effective connectivity. Future studies should clarify the influence of modulating M1 excitability on the effective connectivity from ipsilesional to contralesional M1, which might have clinical significance for facilitating motor recovery in chronic stroke patients.

## Data Availability

The datasets analyzed in this manuscript are not publicly available. Requests to access the datasets should be directed to chunshuiyu@tmu.edu.cn.

## Ethics Statement

This study was approved by the Ethics Committee of Tianjin Medical University General Hospital and informed consent was obtained from each participant before the study. All participants in this study provided written informed consent.

## Author Contributions

YP and MH analyzed the fMRI data and the relationships between the DTI data and fMRI data. YP, ML, and CY wrote the paper. JL analyzed the DTI data. ML and CY designed the study. All authors approved the final version of the paper to be published.

### Conflict of Interest Statement

The authors declare that the research was conducted in the absence of any commercial or financial relationships that could be construed as a potential conflict of interest.
